# Research Advances in the Correlation between Peroxisome Proliferator-Activated Receptor-*γ* and Digestive Cancers

**DOI:** 10.1155/2018/5289859

**Published:** 2018-02-01

**Authors:** Shuqi Xu, Xuanfu Xu

**Affiliations:** Department of Gastroenterology, Shidong Hospital of Shanghai, Anhui Medical University, Shanghai 200433, China

## Abstract

Peroxisome proliferator-activated receptor-*γ* (PPAR*γ*) is a class of ligand-activated nuclear transcription factors, which is a member of type II nuclear receptor superfamily. Previous studies demonstrate that PPAR*γ* is expressed in a variety of tumor tissues and is closely associated with the proliferation and prognosis of digestive system tumors by its roles in mediation of cell differentiation, induction of cell apoptosis, and inhibition of cell proliferation.

## 1. Introduction

As a class of ligand-activated cytokine of steroid hormone receptor, peroxisome proliferator-activated receptor (PPAR) is activated by binding to peroxisome proliferator, which in turn initiates a series of regulatory effects of transcription, thereby participating in adipogenesis, lipid metabolism, metabolic homeostasis, inflammation, tumorigenesis, and its progression [1, 2]. To date, three receptor subtypes of PPAR (*α*, *β*/*δ*, and *γ*) have been found, among which PPAR*γ* is the most widely studied and is expressed in adipose tissues, esophagus, gastrointestinal, liver, spleen, pancreas, and so on. Recently, it has been discovered that PPAR*γ* is closely linked to digestive system tumors, as it is overexpressed in esophageal cancer, gastrointestinal cancer, liver cancer, pancreatic cancer, and para-carcinoma tissues [3–5]. In this paper, we introduce the structure, organization, distribution, ligands, and function of PPAR*γ* briefly and review the correlation between PPAR*γ* receptor and digestive system tumor.

## 2. Overview of PPAR*γ*

### 2.1. Structure of PPAR*γ*

PPAR*γ* gene is located in human's 3p25 chromosome, and according to promoter, exon, and splicing type, PPAR*γ* mRNA has 4 distinct spliceosomes, namely, PPAR*γ*1, PPAR*γ*2, PPAR*γ*3, and PPAR*γ*4. The protein-coding sequence of PPAR*γ*2 mRNA is different from that of the other three spliceosomes, as PPAR*γ*2 has another 30 amino acid residues at the amino terminus. PPAR*γ* can translate into two different proteins, PPAR*γ*1 and PPAR*γ*2, and their biological roles and ways of expression are not the same. There are four functional domains in the structure of PPAR*γ* molecule: the first one is a non-ligand-dependent transcriptional activation domain for the amino terminus, which is a regulatory region with phosphorylation binding sites. It regulates the activity of PPAR*γ* by altering the affinity of the receptor or the ligand via its phosphorylation. The second one is a DNA binding domain (DBD) consisting of two zinc finger structures, which is a binding domain initiating and regulating the gene transcription after binding to the peroxisome proliferator response element. The third one is a regulatory domain with transcriptional activity, and a number of factors in the nucleus regulate the activity of PPAR*γ* via binding to this domain; the fourth one is a ligand binding domain (LBD) at the carboxy-terminus of PPAR*γ*, and PPAR*γ* exhibits its regulation on target gene expression and downstream effects on the binding of the ligand and this structural domain [6, 7].

### 2.2. Tissue Distribution of PPAR*γ*

PPAR*γ* is widely distributed in adipose tissues, esophagus, gastrointestinal, liver, and pancreas, and it is also expressed in various tissues of the immune system. The distribution and range of different types of PPAR*γ* spliceosomes vary in different body tissue cells [8] with high tissue specificity: PPAR*γ*1 is widely distributed throughout the body with different degrees of expression; PPAR*γ*2 is mainly distributed in adipose tissues and liver tissues, with the former expressing more; PPAR*γ*3 is mainly distributed in adipocytes, macrophages, and colonic epithelial cells, where it is also expressed. The distribution of PPAR*γ*4 is not yet clear and remains to be explored.

### 2.3. PPAR*γ* Ligands

The current studies show that the ligand treatment of PPAR*γ* inhibits the proliferation of tumor cells and induces tumor cell apoptosis, which underlines its role in tumor targeted therapy [9, 10]. Currently, a series of PPAR*γ* agonists (also known as PPAR*γ* ligands) have been found or synthesized, which may be further divided into natural and synthetic ligands on the basis of their sources [11]. The natural ligand consists of a group of endogenously secreted molecules, whose activity is often not high. Natural ligands include all kinds of unsaturated fatty acids and their metabolic derivatives, such as linoleic acid, linolenic acid, and eicosapentaenoic acid. Certain prostaglandins and their metabolic derivatives also belong to natural ligands, and currently 15-deoxygenated prostaglandin is known to possess the strongest metabolic activity [12]. On the other hand, the synthetic ligand is mainly composed of thiazolidinedione compounds (troglitazone, rosiglitazone, pioglitazone, etc.). With stronger metabolic activity than that of the natural ligand, the synthetic ligand is widely used in diabetes management. Moreover, a growing body of research has discovered that the synthetic ligand has an antitumor effect either used independently or combined with other medications, and the mechanism underlying this is a research hotspot at present [13]. Certain nonsteroidal drugs, such as indomethacin and ibuprofen, are reported to possess an antineoplastic effect, although their metabolic activity is extremely low [14]. Additionally, some receptor antagonists, such as leukotrienes, also belong to PPAR*γ* agonists.

## 3. Function of PPAR*γ*

PPAR*γ* is widely present in various tissue cells and has a broad array of biological functions. It is involved in the regulation of carbohydrate metabolism and adipogenesis in cells and also participates in the inflammatory response as well as the differentiation and apoptosis of tumor cells [15]. Researchers has found that after being activated by ligand, PPAR*γ* can induce tumor cell differentiation, repress their proliferation, promote their apoptosis, and concomitantly reduce neoplastic angiogenesis, which eventually halts the tumor growth, proliferation, infiltration, and metastasis [16, 17]. Its most important function is mediation of gene transcription and subsequent regulation on its activation after combining with its ligands. The process is briefed as follows: the ligand-activated PPAR*γ* forms a heterodimer complex with 9-cis-retinoid X receptor, retinoid X receptor (RXR), or glucocorticoid receptor, which subsequently forms a receptor-coregulatory factor-DNA or a protein-DNA complex after combining with peroxisome proliferator response element (PPRE, the upstream target gene promoter of PPAR*γ*). As such, a series of PPAR*γ*-mediated molecular events activates the target gene and plays a role in its transcription and regulation. In the course of this process, a series of auxiliary activators or inhibitors can affect the function of PPAR*γ* [18]. PPAR*γ* can also affect gene transcription and regulation by influencing the signal pathways of other specific transcription factors.

## 4. PPAR*γ* and Digestive Tumors

Tumorigenesis involves cell proliferation dysregulation, abnormal differentiation, and apoptosis imbalance. PPAR*γ* is highly expressed in multiple gastrointestinal cancers and is linked to the development, growth, and proliferation of them [19, 20], which again plays an important biological role in halting their invasion and metastasis [21]. Recent studies have shown that PPAR*γ* receptor agonists inhibit the proliferation of various types of tumor cells in vitro and vivo, has a synergistic effect with other antitumor chemotherapeutics [22, 23], and also enhances radiotherapy sensitivity [24]. Currently, PPAR*γ* is used as a potential diagnostic and prognostic biomarker of digestive tract cancers and has become the research focus as a promising therapeutic target for gastrointestinal cancers.

### 4.1. PPAR*γ* and Esophageal Cancer

Esophageal cancer often originates from esophageal squamous intraepithelial neoplasia. PPAR*γ* is expressed in normal esophageal squamous epithelium, esophageal squamous intraepithelial neoplasia, and esophageal squamous cell carcinoma. It is found [25] that the expressions of PPAR*γ* mRNA and protein in the esophageal squamous intraepithelial neoplasia and esophageal squamous cell carcinoma are significantly lower than those of the normal esophageal squamous epithelium and are correlated with the cellular differentiation levels. In esophageal squamous cell carcinoma with various differentiation levels, the expression of PPAR*γ* mRNA and protein is also different, and it has proved that the degree of differentiation of tumor cells is inversely correlated to PPAR*γ* mRNA and protein expression. Taken together, the gene expression of PPAR*γ* is associated with the development and prognosis of esophageal cancer. Since PPAR*γ* is highly expressed in the esophageal cells, PPAR*γ* agonist drugs, such as rosiglitazone, are found to decrease the growth, proliferation, invasion, and metastasis of the cell lines of the esophageal squamous cell carcinoma and act in a certain dose-dependent manner [26]. Such an inhibitory effect is also inversely correlated to the differentiation level of the esophageal squamous cell carcinoma [26]. Besides, some results suggest that efatutazone, but not the conventional PPAR-*γ* agonist troglitazone, alone or in combination with cetuximab, may offer therapeutic effects [27]. At present, it is assumed that after ligand activation, PPAR*γ* inhibits the proliferation and growth of tumor cells in a dose-dependent manner, which is also associated with the differentiation level of tumor cells, suggesting that PPAR*γ* may be an early diagnostic factor for esophageal cancer, which provides clinicians with a new interventive target for esophageal cancer.

### 4.2. PPAR*γ* and Gastric Cancer

Recent studies [28] on PPAR*γ* and gastric cancer show that the expression rate of PPAR*γ* is closely related to the dysplasia of gastric mucosal tissue: the expression level of PPAR*γ* is low in normal gastric mucosa, and the expression of PPAR*γ* in gastric mucosal tissue is significantly lower in patients with chronic atrophic and chronic nonatrophic gastritis than that in patients with atypical hyperplasia of gastric mucosa, while there is a significant high expression in the gastric cancer tissue, indicating that the expression rate of PPAR*γ* in the gastric mucosa is positively correlated to the degree of dysplasia.* Helicobacter pylori* infection is one of the most common carcinogenic factors, and elimination of* Helicobacter pylori* has also reduced the expression of PPAR*γ* in the gastric mucosa, suggesting that PPAR*γ* has been involved in the carcinogenic process of* Helicobacter pylori* [29].

Several papers [30, 31] have reported an antitumor effect of PPAR*γ* agonists (such as thiazolidinedione, prostaglandin, and their metabolic derivatives) in gastric cancer tissues, where PPAR*γ* agonists disturb the growth cycle of tumor cells, induce tumor cell differentiation [28], promote tumor cell apoptosis, repress tumor cell proliferation and metastasis [32], and reduce tumor angiogenesis, and these effects are dose-dependent. The combination of retinoid X receptor agonist (9-cis-retinoic acid) and troglitazone could induce the maximal inhibitory effects on tumor growth and apoptosis via promoting the formation of RXR/PPAR*γ* heterodimer [33].

The research mentioned above shows that the high expression of PPAR*γ* may be the molecular marker of chronic gastritis developing into gastric mucosal atypical hyperplasia or even gastric cancer, and it is expected to be a marker for early detection of gastric cancer and assessment of the malignancy degree. After being activated by ligands, PPAR*γ* can not only inhibit the proliferation of gastric cancer growth, but also prevent the development and growth of gastric cancer. In conclusion, PPAR*γ* may become a new therapeutic target for the gastric cancer treatment.

### 4.3. PPAR*γ* and Colorectal Cancer

PPAR*γ* is expressed in normal intestinal mucosa, colorectal cancer tissues, and paraneoplastic tissues, and the level of PPAR*γ* expression is related to the tumor cell differentiation, infiltration, and metastasis [34, 35]: (1) the higher the differentiation level of colorectal cancer tissue, the higher the expression level of PPAR*γ* protein; (2) the protein expression level of PPAR*γ* is positively related to the range of the infiltration, lymph node metastasis, or distant metastasis. PPAR*γ* agonists can halt the tumor cell growth cycle of the colorectal cancer, induce the differentiation of intestinal cancer cells, change the morphology of tumor cells, and promote tumor cell apoptosis to achieve the antitumor effect [36]. Currently, troglitazone is considered to enhance the apoptotic response of colon cancer cells to photodynamic therapy [37]. Rosiglitazone, as a novel radiosensitizer, suppresses radiation-induced survival signals and DNA damage response and enhances the radiation-induced apoptosis signaling cascade [38]. The combination of resveratrol with a PPAR*γ* agonist could play a role in resveratrol-induced apoptosis of colon carcinoma cells [39]. There are studies [40, 41] indicating that several kinds of PPAR*γ* agonists, such as thiazolidinediones, are expected to combine with chemotherapeutic agents or other target medications to work as a second-line treatment regimen, in the hope of improving the prognosis of patients with advanced colorectal cancer. Conclusively, PPAR*γ* is closely associated with the occurrence, development, proliferation, and prognosis of colorectal cancer and may become a novel molecular biomarker for clinical staging of colorectal cancer and a promising therapeutic target for colorectal cancer.

### 4.4. PPAR*γ* and Liver Cancer

PPAR*γ* is expressed in normal hepatocytes, nonalcoholic fatty liver cells, hepatocellular carcinoma tissues, and various liver cancer cell lines. Studies [42] have shown that, compared to patients with overexpression of PPAR*γ*, patients with low expression of PPAR*γ* have larger primary tumors, more inflicted lymph nodes, and commoner distant metastasis and are often accompanied by vascular invasion. A series of current papers [43] indicate that, due to the differences in metabolic environment, cell types, and carcinogenic signal pathways, PPAR*γ* may be expressed as either inhibiting or promoting the growth and proliferation of the hepatocellular carcinoma. More studies [44] have suggested that after being treated with PPAR*γ* agonists, such as rosiglitazone, the growth of liver cancer cells in vitro can be blocked at Phase G1. Additionally, PPAR*γ* agonists also decrease the specific enzyme for liver tumor cell differentiation (*γ*-glutamyl transferase) and the protein markers (alpha-fetoprotein) significantly. Furthermore, the sensitivity of chemotherapy against liver cancer has been potentiated with rosiglitazone. However, there is a study indicating that rosiglitazone has a pleiotropic anticancer effect independent of PPAR*γ* [45]. An experimentation on animals indicate that, in PPAR*γ*-expressing and PPAR*γ*-deficient mouse models of hepatic carcinogenesis, PPAR*γ* deletion in hepatocytes of did not modify hepatic carcinogenesis but increased the thiazolidinedione antitumorigenic effect in part by inhibition of nucleophosmin expression and p53 activation [46]. We hypothesize that the net effect of thiazolidinedione in cancer cells depends on the balance of PPAR*γ*-mediated (prooncogenic) and PPAR*γ*-independent (antioncogenic) mechanisms, by means of regulation different factors including receptor expression levels, phosphorylation status, expression of the heterodimeric partners, and the presence of endogenous ligands.

Overall, the studies mentioned above suggest that, at present, the role of PPAR*γ* in the formation, differentiation, proliferation, and apoptosis of hepatocellular carcinoma cells is still unclear, and the regulation role of PPAR*γ* in energy metabolism and adipogenesis as well as its relation to hepatocellular carcinoma should be further investigated.

### 4.5. PPAR*γ* and Pancreatic Cancer

**Figure 1:**
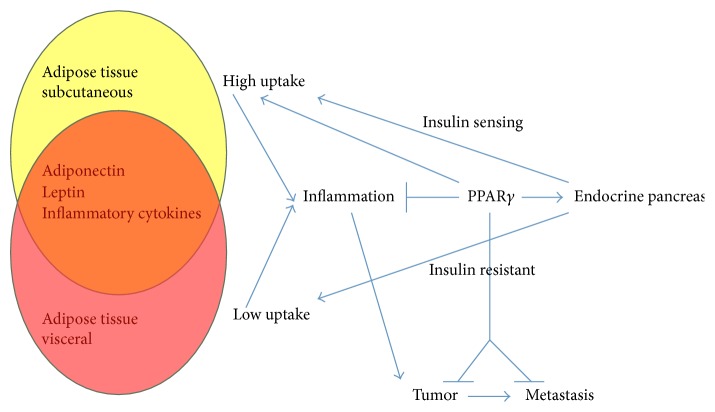
Effects of peroxisome proliferator-activated receptor-*γ* on diabetes, obesity, and pancreatic ductal adenocarcinoma [5].

PPAR*γ* is expressed in normal pancreas, pancreatic cancer, and para-carcinoma tissues and is highly expressed in pancreatic cancer tissue cells. Insulin resistance, obesity, oxidative stress, and inflammatory response all play important roles in pancreatic cancer (Figure 1). PPAR*γ* indirectly regulates the insulin transcription mediated by pancreatic and duodenal homeobox 1 (PDX-1), who is a master regulator of pancreas development and differentiation and regulates the expression of insulin gene in *β*-cells [47]. Through the regulation of the cholesterol transporter ATP-binding cassette transporter A1, PPAR*γ* regulates insulin secretion regulating the expression of G-protein-coupled transmembrane receptor [48]. By means of increasing the uptake of glucose in the skeletal and adipose tissue by regulation of glucose transporters, also promoting catabolic events to produce ATP by increasing insulin signaling modulating AMP-activated protein kinase (AMPK), PPAR*γ* increases the insulin sensitivity in the peripheral tissues [49]. PPAR*γ* is a master regulator of adipocytes differentiation. By the Wnt/*β*-catenin pathway that recruits the Single Molecule, Real-Time (SMRT) corepressor complex to repress PPAR*γ*1 and PPAR*γ*2 gene, preadipocytes are maintained in undifferentiated state [50]. In preadipocytes, activation of PPAR*γ* leads to the novo differentiation of subcutaneous adipocytes and the apoptosis of older visceral adipocytes [51]. Moreover, in the new adipose tissue, PPAR*γ* reduces leptin expression indirectly by antagonism on leptin promoter with CCAAT/enhancer binding protein (C/EBP), whereas the nuclear receptor transcription factors control adiponectin directly which also induces the secretion of HMW adiponectin from adipocytes [52]. In adipocytes, PPAR*γ* activation has been associated with the upregulation of insulin receptor substrate- (IRS-) 2 and cytapheresis components of insulin pathway and hence with increased insulin sensitivity [53]. PPAR*γ* inhibits resistin synthesis, which is an adipokine associated with inflammation and diabetes type 2, which is the main source of macrophages [54]. Depending on the stimuli of the environment, macrophages can acquire distinct phenotypes: the M1 is an inflammatory phenotype, whereas the M2 is anti-inflammatory. In obese and diabetes type 2 patients, adipose tissue is particularly rich in M1 macrophages. PPAR*γ* primes primary human monocytes into M2 differentiation and keeps M2 marker expression in resting state or M1 macrophages [55]. In adipose tissue, PPAR*γ* reduces the production of the proinflammatory cytokines tumor necrosis factor- (TNF-) *α*, interleukin- (IL-) 6, and plasminogen activator inhibitor- (PAI-) 1. Suppression of these cytokines is associated with the repression of nuclear factor kappa-light-chain-enhancer of activated B cells (NF-*κ*B) signaling cascade. PPAR*γ* transrepresses NF-*κ*b acting as a transcriptional corepressor of NF-*κ*B-target genes or by direct binding with NF-*κ*B; the nuclear receptor may reduce NF-*κ*B activation and induce its degradation [56, 57]. In immune cells, the inhibition of NF-*κ*B signaling by PPAR*γ* results in a substantial anti-inflammatory response [57].

Since PPAR*γ* is involved in carbohydrate metabolism, adipogenesis, and inflammation, PPAR*γ* agonists have been widely used in the management of diabetes and oxidative stress-related diseases, and its antineoplastic effect against pancreatic cancer has been further confirmed in a series of studies [5]. Some studies propose that a triple-agent regime, including metformin, pioglitazone, and lithium, may provide a metabolic adjuvant therapy for pancreatic cancer [58]. In the development and progression of pancreatic cancer, the activated PPAR*γ* inhibits tumor cell proliferation and growth and promotes tumor cell apoptosis by inducing cell differentiation, regulating cell cycle and mediating the expression of target genes including apoptotic genes and proapoptotic genes. Altogether, PPAR*γ* has the antitumor effect through a series of complicate biological functions. This combination of interferon-*β* and the PPAR-*γ* agonist troglitazone induced a synergistic effect on the growth inhibition of BxPC-3, a pancreatic cancer cell line, through the counteraction of the interferon-*β*-induced activation of signal transducer and activator of transcription- (STAT-) 3, the mitogen-activated protein kinase (MAPK), and AKT and the increase in the binding of both STAT-1 related complexes and PPAR-*γ* with specific DNA responsive elements [59]. Some papers have suggested [58, 60] that prostaglandin and PPAR*γ* activated by its metabolic derivatives inhibit the growth and proliferation of pancreatic cancer cells in a certain dose-dependent manner; rosiglitazone and metformin, either used alone or combined, both inhibit the growth and proliferation of pancreatic cancer in vitro and promote the tumor cell apoptosis, and it has been found that the potency of their combination is stronger than that of either single drug. Consequently, it is indicated that PPAR*γ* may provide a new molecular biological marker for the early diagnosis of pancreatic cancer and also offer new ways to reinforce the therapy effect of pancreatic cancer and improve its prognosis.

## 5. Conclusions and Prospects

At present, the incidence and mortality rate of gastrointestinal cancer in China are relatively high, whose pathophysiological mechanisms are also relatively complex. The development and progress are a sophisticate multistep, multistage process involving a variety of genes. By the time of treatment, cancer invasion and metastasis have occurred in the majority of patients with gastrointestinal cancer, which makes the efficacy of surgery, radiotherapy, and chemotherapy poor. Therefore, it will be of great significance to look for a new molecular marker of digestive tumors in order to assist their diagnosis and to explore new targets for the targeted intervention.

In summary, PPAR*γ* is closely related to digestive system tumors. After being activated by the ligand, it can inhibit the development and proliferation of tumor cells through a series of molecular biological effects, such as induction of tumor cell differentiation, promotion of tumor cell apoptosis, inhibition of tumor cell proliferation, and the invasion as well as metastasis, while its detailed molecular biological mechanism still needs an in-depth study. With regard to the diagnosis and treatment of gastrointestinal cancer, PPAR*γ* has now become a molecular biology marker for the detection of gastrointestinal tumors as well as a molecular star in target therapy for gastrointestinal cancer, which may prove a breakthrough in the diagnosis and management of digestive system cancers.
